# NRF2-Targeted Therapy in Cardiovascular Disease Transitions from Systemic Activation to Precision Redox Medicine

**DOI:** 10.3390/antiox15020219

**Published:** 2026-02-08

**Authors:** Yizhao Peng, Jinhong Wei, Yang Yang

**Affiliations:** School of Life Sciences and Medicine, Northwest University, Xi’an 710069, China

**Keywords:** NRF2, oxidative stress, cardiac remodeling, heart failure, precision redox medicine

## Abstract

The transcription factor Nuclear Factor Erythroid 2-Related Factor 2 (NRF2) governs cellular redox homeostasis and serves as a primary defense mechanism against oxidative stress-driven cardiac remodeling. Beyond basal antioxidant effects, NRF2 coordinates a broad defensive network that preserves mitochondrial bioenergetics, maintains proteostasis, and inhibits regulated cell death pathways, including necroptosis and ferroptosis. Despite robust efficacy in preclinical models, translating these findings to the clinic remains challenging. This review examines the molecular structure of the NRF2-KEAP1 axis, synthesizing evidence regarding its efficacy in ischemia–reperfusion injury and diabetic cardiomyopathy, while assessing the mechanisms of pathway repression and the liabilities of indiscriminate activation. We further review different pharmacological strategies, contrasting the clinical limitations of electrophiles with the potential of protein–protein interaction inhibitors. Finally, we discuss innovations such as cardiac-targeted delivery and biomarker-guided stratification, critically assessing whether these approaches can overcome safety barriers and emphasizing that rigorous validation is essential for clinical viability.

## 1. Introduction

Cardiovascular disease (CVD) remains the leading cause of global mortality, with heart failure (HF) representing its terminal common pathway. Despite the expansion of guideline-directed medical therapies, the prevalence of HF, particularly heart failure with preserved ejection fraction (HFpEF), continues to rise [1,2]. The progression to HF is driven by pathological cardiac remodeling, a maladaptive process characterized by cardiomyocyte hypertrophy, fibroblast activation, and interstitial fibrosis. These structural and functional alterations compromise myocardial mechanics and metabolic flexibility, ultimately leading to pump failure [3].

The myocardium, characterized by its high metabolic demand and limited regenerative capacity, is uniquely dependent on strict redox homeostasis. Unlike the liver, the heart possesses relatively low constitutive antioxidant reserves, making it disproportionately susceptible to redox perturbations. Physiologically, reactive oxygen and nitrogen species (ROS/RNS) act as essential second messengers, modulating protein function via reversible post-translational modification of thiol groups on key signaling molecules involved in excitation-contraction coupling and autophagy [4,5]. However, in disease states, the precision of this signaling network is compromised. The transition to oxidative stress is not simply an excess of ROS, but a disruption of spatial and temporal signaling specificity [6]. In this state, ROS cause macromolecular damage and activate pathological signaling cascades that promote cardiac dysfunction. In the failing heart, oxidative stress is sustained by crosstalk between multiple ROS sources. Mitochondrial dysfunction is a primary driver, generating ROS via mechanisms such as reverse electron transport (RET), particularly under conditions of substrate overload [7]. Simultaneously, neurohormonal and biomechanical stressors upregulate nicotinamide adenine dinucleotide phosphate (NADPH) oxidase isoforms (NOX2 and NOX4), which interact with mitochondria to perpetuate ROS production [8]. Additionally, the depletion of the cofactor tetrahydrobiopterin (BH4) leads to the uncoupling of endothelial nitric oxide synthase (eNOS), shifting its enzymatic function to produce superoxide instead of nitric oxide (NO) [9]. These interactions establish a feed-forward mechanism of ROS-induced ROS release, maintaining a pro-oxidant state.

Critically, oxidative stress does not act in isolation but serves as a proximal trigger that precipitates a cascade of downstream deleterious events, linking redox dysregulation to inflammation, cell death, and fibrosis. In terms of inflammatory coupling, excessive ROS promotes the leakage of mitochondrial DNA (mtDNA) into the cytosol, which functions as a damage-associated molecular pattern (DAMP) to activate the nucleotide-binding oligomerization domain-like receptor family pyrin domain-containing 3 (NLRP3) inflammasome and nuclear factor kappa B (NF-κB) signaling, thereby fueling sterile inflammation [10]. Concurrently, mitochondrial ROS (mtROS) overload initiates lethal signaling cascades by triggering the opening of the mitochondrial permeability transition pore (mPTP) and subsequent cytochrome c release, driving both intrinsic apoptotic and necroptotic pathways [11,12]. In the cardiac interstitium, redox imbalance exacerbates structural remodeling by promoting myofibroblast differentiation via the transforming growth factor-beta (TGF-β)/Smad axis and activating matrix metalloproteinases (MMPs) that degrade the extracellular matrix [13]. Therefore, the therapeutic rationale for targeting antioxidant systems extends beyond simple radical scavenging, it represents a strategy to sever the upstream link between redox dysregulation and this broad spectrum of maladaptive processes.

The primary cellular defense against such proteotoxic and oxidative insults is the NRF2 pathway [14]. Under basal conditions, NRF2 is sequestered and targeted for ubiquitin-mediated degradation by Kelch-like ECH-associated protein 1 (KEAP1). Upon exposure to electrophilic or oxidative stress, cysteines within KEAP1 are modified, halting degradation and allowing NRF2 to translocate to the nucleus. There, it binds the antioxidant response element (ARE) to drive a cytoprotective gene network. Significantly, NRF2 is not solely an antioxidant transcription factor but serves as a master regulator of cellular homeostasis. It coordinates a comprehensive program governing xenobiotic detoxification, proteostasis via autophagy and proteasome subunits, mitochondrial biogenesis, and metabolic reprogramming, including NADPH generation [15]. Thus, the NRF2-KEAP1 axis functions as a central integration hub for stress signals, influencing cell survival and the trajectory of remodeling. This review evaluates the roles of NRF2 in cardiovascular pathophysiology, dissecting its regulatory network and discussing the context-dependent challenges of targeting this pathway in heart disease.

## 2. The NRF2-KEAP1 Axis

### 2.1. Molecular Structure and Regulatory Mechanisms

#### 2.1.1. NRF2-KEAP1 Molecular Structure

Under homeostatic conditions, the NRF2 transcription factor exhibits a high turnover rate, a feature critical for its rapid inducibility. This dynamic instability is orchestrated by its direct repressor, KEAP1, which functions as a substrate adaptor for a Cullin 3 (CUL3)-RING-box 1 (RBX1) E3 ubiquitin ligase complex [16]. Structurally, the regulation relies on the N-terminal Neh2 domain of NRF2, which contains two distinct binding motifs known as the high-affinity ETGE motif and the lower-affinity DLG motif. These motifs engage the Kelch domains of the KEAP1 homodimer in a coordinated hinge-and-latch mechanism. This configuration optimally positions NRF2 for polyubiquitination at specific lysine residues within the Neh2 domain, thereby targeting it for degradation by the 26S proteasome. Consequently, this continuous cycle maintains NRF2 at low basal levels, ensuring the system remains poised for immediate activation upon stress exposure [17].

#### 2.1.2. Stress Induced Activation

KEAP1 acts as a critical intracellular redox sensor containing multiple highly reactive sentinel cysteine residues, such as C151, C273, and C288 in the human protein [18]. Covalent modification of these thiols by oxidants or electrophiles triggers a conformational change in the KEAP1 homodimer, disrupting the lower-affinity latch interaction formed by the DLG motif. This structural alteration compromises the ability of the E3 ligase complex to ubiquitinate NRF2, allowing newly synthesized NRF2 to escape degradation, accumulate in the cytoplasm, and translocate to the nucleus [19]. However, this classic activation represents only one facet of a broader regulatory network. Crucial layers of fine-tuning are provided by classic pathways, such as selective autophagy mediated by the autophagy receptor p62/SQSTM1. This protein can target KEAP1 for degradation, thereby liberating NRF2 and creating a powerful positive feedback loop since p62 is itself a prominent NRF2 target gene [20]. Furthermore, post-translational modifications by various protein kinases, including protein kinase C (PKC), glycogen synthase kinase-3 beta (GSK-3β), and protein kinase R-like endoplasmic reticulum kinase (PERK), can phosphorylate NRF2 or KEAP1 to modulate stability and nuclear localization. Recent evidence also points to the formation of stress induced liquid–liquid phase separated (LLPS) condensates containing p62 and KEAP1, suggesting that regulation occurs within specialized, dynamic subcellular compartments [21]. This multimodal regulation allows the cell to integrate diverse stress signals beyond simple redox changes.

#### 2.1.3. Transcriptional Targets

**Figure 1:**
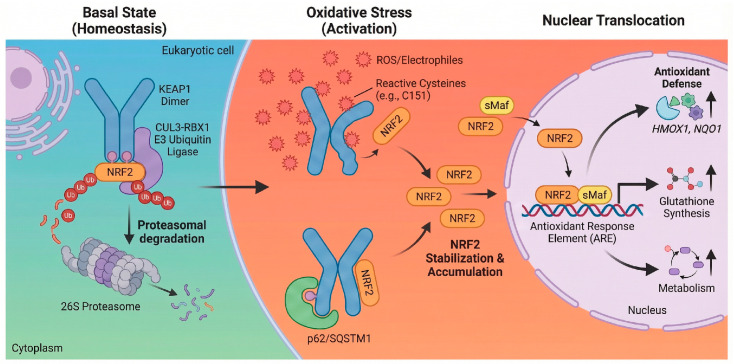
Schematic of the NRF2-KEAP1 signaling pathway. Basal state: Under homeostatic conditions, KEAP1 binds NRF2 in the cytoplasm and serves as an adaptor for the CUL3-RBX1 E3 ubiquitin ligase complex, targeting NRF2 for proteasomal degradation [16]. Oxidative stress: ROS or electrophiles modify reactive cysteine residues on KEAP1 (e.g., C151), causing a conformational change [18]. This inhibits ubiquitination, allowing NRF2 to accumulate. Non-classic mechanisms, such as p62/SQSTM1-mediated autophagy, also contribute to NRF2 stabilization [20]. Nuclear translocation: Stabilized NRF2 translocates to the nucleus and heterodimerizes with sMaf. The complex binds to ARE to induce the transcription of genes regulating antioxidant defense (HMOX1, NQO1), glutathione synthesis, and metabolism [20,22]. Created in BioRender. Peng, Y. (2025) https://BioRender.com/xh710le (accessed on 3 February 2026).

Once in the nucleus, NRF2 forms obligate heterodimers with small Maf (sMaf) proteins such as MafG and MafK. This dimerization is essential for high-affinity binding to the cis-acting regulatory sequence known as the ARE, characterized by the core consensus sequence 5′-TGACnnnGC-3′ [22]. This binding event initiates a multi-layered transcriptional program designed to restore cellular homeostasis across several functional domains [14]. Foremost among these is the upregulation of antioxidant and detoxification enzymes, including heme oxygenase-1 (HMOX1) and NADPH quinone dehydrogenase 1 (NQO1), which reinforce antioxidant defenses and modulate redox homeostasis to counter ROS accumulation. Simultaneously, NRF2 coordinates the induction of thiol-based redox buffers, regulating genes for glutathione biosynthesis (GCLC, GCLM), regeneration (GSR), and utilization (GSTs), as well as components of the thioredoxin system (TXN, TXNRD1) [14,22]. Beyond direct scavenging, the pathway drives metabolic reprogramming to support these defenses, notably by redirecting glucose flux into the pentose phosphate pathway via glucose-6-phosphate dehydrogenase (G6PD) to generate the reducing equivalent NADPH. Furthermore, NRF2 enhances proteostasis by increasing the expression of proteasome subunits and autophagy-related genes like p62/SQSTM1, facilitating the clearance of damaged proteins and organelles [14,23]. Finally, the pathway exerts potent anti-inflammatory effects by suppressing pro-inflammatory gene expression, such as interleukin-6 (IL-6) and interleukin-1 beta (IL-1β), often by interfering with NF-κB signaling [24]. This coordinated response underscores the role of NRF2 not merely as an antioxidant factor but as a central regulator of cellular defense [14,25], as illustrated in Figure 1.

### 2.2. NRF2-Mediated Redox Defense in Different Cardiac Cell Types

#### 2.2.1. Cardiomyocytes

In the high energy demand environment of cardiomyocytes, NRF2 is critical for maintaining mitochondrial function and cell survival. Its activation directly counteracts mtROS, preserving the integrity of the electron transport chain and preventing the opening of the mPTP-an irreversible step in cell death pathways such as apoptosis and necroptosis [26]. Beyond simple ROS scavenging, NRF2 orchestrates mitochondrial quality control by promoting mitochondrial biogenesis partly through the transcriptional upregulation of *Ppargc1a* (encoding PGC-1α) and facilitating the autophagic clearance of damaged mitochondria, known as mitophagy [27]. This ensures a healthy and efficient mitochondrial pool, sustaining the high ATP production required for cardiac contraction. Furthermore, by improving bioenergetics and mitigating damage to ion channels and calcium handling proteins like SERCA2a, NRF2 helps preserve contractile function and prevent arrhythmogenesis [28].

#### 2.2.2. Cardiac Fibroblasts

NRF2 acts as a negative regulator of pathological cardiac fibrosis. ROS function as critical cofactors for the activation of the master pro-fibrotic cytokine, transforming growth factor-β1 (TGF-β1). NRF2-driven antioxidant activity scavenges these ROS, raising the threshold for fibroblast activation. Additionally, NRF2 antagonizes TGF-β1/Smad signaling by promoting Smad7 expression or interfering with Smad2/3 nuclear accumulation [29]. By attenuating this fibrogenic axis, NRF2 inhibits the transdifferentiation of fibroblasts into myofibroblasts [30], thereby limiting the deposition of extracellular matrix proteins. This preservation of myocardial compliance is essential for preventing diastolic dysfunction and the progression of heart failure.

#### 2.2.3. Vascular Endothelial Cells

**Figure 2:**
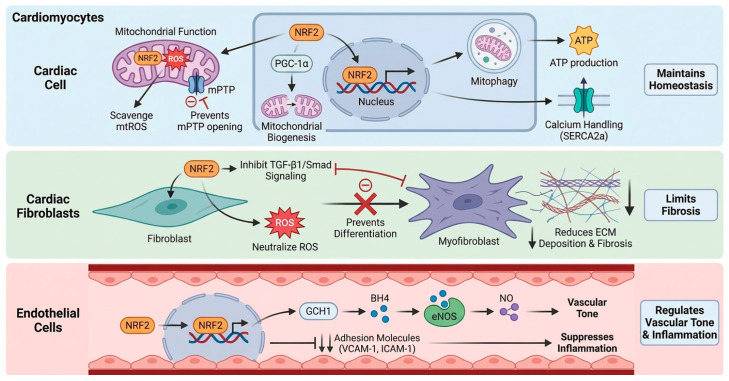
Cell-specific roles of NRF2 in the cardiovascular system. Cardiomyocytes: NRF2 maintains mitochondrial function by scavenging mtROS and preventing mPTP opening [26]. It also promotes mitochondrial biogenesis (via PGC-1α) and mitophagy, ensuring efficient ATP production and calcium handling (e.g., SERCA2a) [27,28]. Cardiac Fibroblasts: NRF2 limits fibrosis by scavenging ROS and inhibiting TGF-β1/Smad signaling [29,30]. This suppression prevents fibroblast-to-myofibroblast differentiation and reduces extracellular matrix (ECM) deposition. Endothelial Cells: NRF2 regulates vascular tone and inflammation. It maintains eNOS coupling via GCH1-mediated BH4 synthesis to ensure NO bioavailability and suppresses the expression of adhesion molecules (VCAM-1, ICAM-1) [31,34]. Created in BioRender. Peng, Y. (2025) https://BioRender.com/v2baujo (accessed on 3 February 2026).

Within the cardiac vasculature, NRF2 is a key regulator of endothelial homeostasis. Its activation maintains eNOS coupling, primarily by upregulating GTP cyclohydrolase I (GCH1) to synthesize the essential cofactor tetrahydrobiopterin (BH4). This prevents eNOS uncoupling and superoxide production, ensuring NO bioavailability and endothelium-dependent vasodilation [31,32]. The NRF2 target gene HMOX1 further contributes to vasodilation via the production of carbon monoxide (CO) [33]. Critically, NRF2 promotes an anti-inflammatory and anti-thrombotic phenotype by suppressing adhesion molecules, such as VCAM-1 and ICAM-1, and pro-inflammatory cytokines, reducing leukocyte recruitment and vascular inflammation [34], as depicted in Figure 2.

#### 2.2.4. Other Cell Populations

Beyond the major cardiac cell types, NRF2 plays a critical role in the immune and conduction compartments. In macrophages, NRF2 activation promotes a phenotypic switch from the pro-inflammatory M1 state to the reparative M2 phenotype, thereby limiting post-infarction inflammation and facilitating tissue repair [35]. Furthermore, within the cardiac conduction system, NRF2 helps maintain the redox state of critical ion channels. ROS-induced oxidation of CaMKII and voltage-gated sodium channels (Nav1.5) provides a substrate for arrhythmias [36]; thus, constitutive NRF2 activity in these cells serves as an intrinsic anti-arrhythmic barrier, preserving electrical stability under stress.

## 3. Pathophysiological Mechanisms of NRF2 in CVD 

The functional impact of NRF2 within the cardiovascular system is not binary but is determined by the nature, duration, and intensity of the stressor. While NRF2 serves a primary antioxidant regulator against acute oxidative insults, its role in chronic remodeling is complicated by impaired signaling capacity, and its excessive activation carries the risk of reductive stress [37].

### 3.1. Regulation of Oxidative Stress and Cell Death During CVD

The rapid mobilization of NRF2 is critical in scenarios of acute oxidative surges, particularly ischemia/reperfusion (I/R) injury. The burst of ROS upon reperfusion overwhelms basal defenses, triggering a cascade of regulated cell death. NRF2-knockout mice exhibit expanded infarct sizes, severe contractile dysfunction, and post-I/R lethality, confirming the essential role of the endogenous pathway [32]. Mechanistically, the protective scope of NRF2 extends beyond simple ROS scavenging to the regulation of distinct cell death modalities. One primary mechanism involves the inhibition of necroptosis, where NRF2 upregulates downstream effectors such as heme oxygenase-1 (HO-1) and NQO1. These enzymes function to preserve mitochondrial membrane potential and inhibit the opening of the mPTP, thereby preventing cytochrome c release and subsequent cell death [38,39,40]. Furthermore, recent evidence highlights a critical role for NRF2 in the suppression of ferroptosis, an iron-dependent form of cell death driven by phospholipid peroxidation. By directly transcriptionally activating SLC7A11 and glutathione peroxidase 4 (GPX4), NRF2 maintains intracellular glutathione levels and reduces lipid hydroperoxides, effectively limiting the propagation of lethal lipid ROS waves during the reperfusion phase [41].

Crucially, NRF2 also plays a pivotal role in stabilizing cardiac electrophysiology. ROS-induced oxidation of CaMKII and sarcolemmal ion channels, such as Nav1.5, predisposes the heart to fatal reperfusion arrhythmias [36]. By maintaining the reduced state of these channels within the cardiac conduction system, NRF2 effectively raises the threshold for arrhythmogenesis [41]. Consequently, pharmacological pre-conditioning with inducers like sulforaphane acts as a protective shield, limiting structural infarction, ferroptotic cell loss, and electrical instability [42].

### 3.2. Modulation of Inflammation and Fibrosis in Chronic Remodeling

In chronic conditions, the landscape changes significantly. NRF2 serves as a critical modulator of cardiac fibrosis and inflammation, under conditions of chronic pressure overload or diabetic cardiomyopathy, NRF2 acts as a negative regulator of fibrosis and hypertrophy [43]. Genetic ablation of NRF2 accelerates the transition to heart failure, a process driven by ROS-dependent pro-fibrotic signaling via the TGF-β/Smad axis and lipid peroxidation [13,39,44,45]. Furthermore, in diabetic models, NRF2 activation specifically dampens NLRP3 inflammasome activity, breaking the cycle between metabolic dysfunction and inflammation [40].

However, a clinically relevant paradox is that NRF2 activity is often significantly blunted in human end-stage heart failure, a state of acquired insufficiency [46]. This phenomenon, termed “acquired NRF2 insufficiency”, implies that the antioxidant reserve is exhausted during the progression from compensated hypertrophy to decompensated failure. The underlying mechanisms constitute a multifactorial blockade [47]. Epigenetic silencing plays a major role, as the NRF2 promoter undergoes hypermethylation by DNA methyltransferases (DNMTs) alongside repressive histone deacetylation mediated by upregulated HDACs, effectively locking the gene in a transcriptionally silent state [48]. This is compounded by aberrant degradation pathways, where chronic neurohumoral stimulation leads to the persistent activation of kinases such as GSK-3β and Fyn, these kinases phosphorylate NRF2 to promote its nuclear export and degradation independent of the KEAP1 pathway [49]. Moreover, metabolic exhaustion ensues, as NRF2 is required for the expression of fatty acid oxidation genes such as CPT1A. Its downregulation forces the failing heart to rely on inefficient glycolysis, creating an energy-deficit cycle that worsens contractile failure. Finally, impaired autophagy disrupts the p62-mediated positive feedback loop, further compromising the cell’s ability to stabilize NRF2 [44]. This deficiency suggests that in chronic settings, the therapeutic goal is not merely activation but the restoration of a dysfunctional signaling machinery [44,45].

### 3.3. Pathological Consequences of Reductive Stress and Hyperactivation

Despite its protective potential, viewing NRF2 activation as a universally beneficial strategy is an oversimplification. Pathological hyperactivation introduces significant risks associated with the “U-shaped” therapeutic window.

The primary risk is reductive stress and protein aggregation [50]. Physiological levels of ROS are essential signaling messengers for processes such as angiogenesis and insulin signaling [5]. The abrogation of these signals by supranormal NRF2 levels results in a highly reducing environment that impairs the formation of disulfide bonds required for protein folding [51]. This can paradoxically induce cardiomyopathy, as observed in models of desmin-related cardiomyopathy, where excessive reduction exacerbates the accumulation of toxic protein aggregates, leading to heart failure [5,51].

Furthermore, the impact of NRF2 is spatially distinct, highlighting a context-dependent maladaptation [5]. While NRF2 loss is detrimental in the left ventricle, its constitutive hyperactivation contributes to the pathology of pulmonary arterial hypertension (PAH) [52]. In the right ventricle and pulmonary vasculature, aberrant NRF2 activation promotes a “cancer-like” pro-proliferative phenotype in smooth muscle cells and fibroblasts, worsening vascular remodeling and right ventricular failure [53]. This stark contrast underscores the complexity of systemic NRF2 targeting.

**Figure 3:**
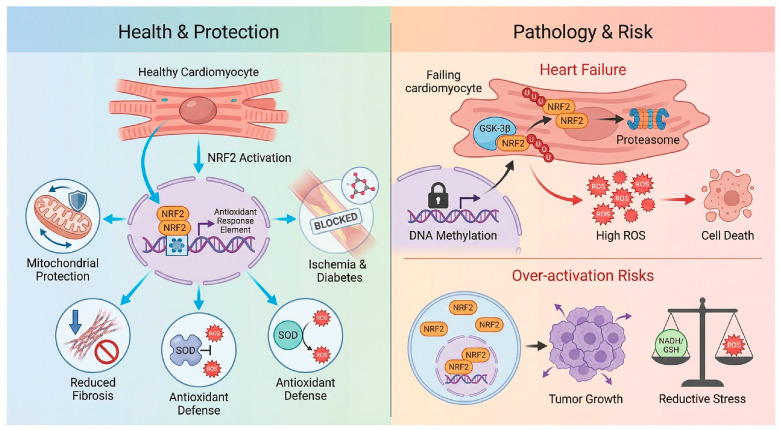
The double-edged sword of NRF2 modulation in cardiovascular disease. Health & Protection: In the context of I/R, pressure overload, and diabetic cardiomyopathy, NRF2 activation exerts cardioprotective effects by scavenging ROS, preserving mitochondrial integrity, and inhibiting inflammation/fibrosis [32,38,39,40]. Pathology & Risk: The therapeutic window is limited by two opposing factors. Top: In advanced heart failure, “Acquired NRF2 Insufficiency” occurs due to epigenetic silencing such as promoter methylation, and enhanced degradation via GSK-3β, rendering the heart susceptible to oxidative damage [43]. Over-activation Risks Conversely, constitutive or systemic NRF2 hyperactivation carries risks of reductive stress, metabolic dysregulation, and the potential acceleration of pre-existing malignancies [13,39,44,45]. Created in BioRender. Peng, Y. (2025) https://BioRender.com/i14v33m (accessed on 3 February 2026).

Beyond cardiac toxicity, systemic NRF2 hyperactivation raises concerns regarding oncogenic mimicry. Somatic gain-of-function mutations in NRF2 or loss-of-function mutations in KEAP1 drive numerous malignancies by facilitating chemoresistance and metabolic reprogramming [53]. Although therapeutic activation has not been directly linked to de novo cardiac tumorigenesis, long-term systemic use of potent inducers could theoretically provide a growth advantage to subclinical, pre-malignant lesions. Additionally, chronic hyperactivation stimulates anabolic pathways, shunting glucose and glutamine toward NADPH generation and biosynthesis, which may disrupt whole-body energy homeostasis and lipid metabolism [25]. Consequently, therapeutic strategies must prioritize temporally controlled and cardiovascular-targeted delivery to avoid tipping the balance from oxidative damage to reductive stress, as shown in Figure 3.

## 4. Therapeutic Targeting of NRF2

The clinical translation of NRF2 modulation has been characterized by a significant inconsistency between preclinical efficacy and clinical outcomes. While animal models consistently demonstrate that NRF2 activation confers cardioprotection against ischemic and non-ischemic injury, human trials utilizing systemic electrophilic activators have highlighted significant safety concerns, most notably the risk of fluid retention and heart failure in vulnerable populations. The therapeutic landscape is currently evolving across three primary approaches: pharmacological innovation, advanced delivery systems, and biomarker-guided stratification. Consequently, current research efforts are focused on addressing these safety liabilities to improve the therapeutic index. The following sections review the evidence regarding current pharmacological strategies.

### 4.1. Pharmacological Strategies

#### 4.1.1. Covalent Electrophilic Modulators

Initial strategies focused on electrophilic compounds that covalently modify reactive cysteines on KEAP1. The prototype of this class is the phytochemical sulforaphane (SFN). While SFN reduces infarct size and fibrosis in rodent models, its clinical translation has been hindered by rapid metabolism via glutathione conjugation and variable oral bioavailability [54,55,56]. To improve potency, synthetic oleanane triterpenoids, such as bardoxolone methyl (CDDO-Me), were developed. In trials for chronic kidney disease (CKD), bardoxolone significantly improved the estimated glomerular filtration rate (eGFR) [57]. However, the pivotal Phase III BEACON trial was terminated early due to a significant increase in heart failure-related adverse events in the treatment arm [58]. Post hoc analyses attributed this outcome not to direct cardiotoxicity, but to fluid retention potentially mediated by off-target modulation of the endothelin-1 pathway [59,60]. Similarly, Dimethyl Fumarate (DMF), while approved for multiple sclerosis, activates NRF2 via succination of KEAP1 but also engages the hydroxycarboxylic acid receptor 2 (HCAR2). This off-target interaction is linked to adverse effects such as cutaneous flushing and gastrointestinal distress [60,61]. For patients with cardiovascular diseases, this systemic limitation is particularly problematic, as they often require long-term treatment and are highly sensitive to hemodynamic fluctuations, this underscores the necessity of developing more cardiac-specific NRF2 modulators or targeted delivery platforms to minimize systemic exposure. These clinical data indicate that while high-potency electrophiles can induce antioxidant gene expression, their systemic reactivity and off-target profiles present substantial safety concerns, particularly for patients with compromised cardiac reserve.

#### 4.1.2. Non-Covalent Protein-Protein Interaction (PPI) Inhibitors

To address the lack of specificity associated with electrophilic cysteine modification, drug discovery has shifted toward the non-covalent disruption of the KEAP1-NRF2 protein–protein interaction (PPI). Small-molecule inhibitors in this class are designed to competitively bind to the Kelch domain of KEAP1, displacing NRF2 without chemically modifying cellular proteins [62]. In preclinical studies, prototypes including naphthalene-based compounds and isoquinoline derivatives have attenuated diabetic cardiomyopathy phenotypes [63]. However, the clinical viability of PPI inhibitors remains unproven. These compounds often face significant challenges regarding solubility and membrane permeability. Moreover, while they may reduce off-target toxicity, evidence is currently lacking regarding their long-term safety profiles. PPI inhibition does not inherently resolve the physiological risk of constitutive NRF2 hyperactivation, suggesting that establishing a precise therapeutic window remains a necessity regardless of the activation modality [15].

#### 4.1.3. Targeted Delivery and Regulated Gene Expression

Minimizing systemic exposure while maximizing cardiac benefit remains a central objective of NRF2 therapy. Nanomedicine offers a potential strategy through the engineering of cardiac-homing nanoparticles. Liposomes or polymeric nanoparticles can be functionalized with ligands such as ischemic-targeting peptides (e.g., CSTSMLKAC), ensuring that NRF2 activators are released preferentially at sites of myocardial injury [64,65]. Beyond synthetic carriers, biological vectors like exosomes-endogenous nanovesicles derived from stem cells-are being explored as biocompatible delivery vehicles for NRF2 mRNA or protein, offering lower immunogenicity. Furthermore, exosome-based interventions are increasingly viewed as a promising therapeutic strategy that may enhance endogenous antioxidant defenses while potentially bypassing some of the safety concerns associated with live-cell therapies. Parallel efforts in gene therapy utilize adeno-associated viruses, specifically the cardiotropic serotype AAV9, to drive NRF2 overexpression directly in cardiomyocytes [66]. To avoid the maladaptive consequences of unchecked expression, next-generation vectors incorporate cardiac-specific promoters (e.g., α-MHC) or inducible Tet-on systems, allowing clinicians to temporally control NRF2 activity [67]. However, it is crucial to acknowledge that these targeted approaches are still in nascent stages. Although they theoretically decouple cardioprotection from systemic toxicity, their clinical safety and efficacy remain unproven. Translating these technological platforms from murine models to human heart failure requires overcoming significant barriers in manufacturing, stability, and potential immunogenicity before they can be considered viable therapeutic options.

### 4.2. The Translational Roadmap

#### 4.2.1. Defining the Therapeutic Window

A critical barrier to clinical success is the definition of the optimal therapeutic window. This requires a nuanced understanding of dose, timing, and duration. While preclinical data suggest that pulsatile, transient activation during acute stress (e.g., post-MI reperfusion) may be superior to chronic, low-level modulation, this hypothesis has not yet been validated in humans. Chronic activation carries the theoretical risk of inducing reductive stress or metabolic inflexibility. Therefore, rather than assuming the efficacy of continuous dosing, future investigations should evaluate whether “hit-and-run” strategies tailored to the distinct phases of cardiac remodeling offer a superior safety profile [15].

#### 4.2.2. Combination Therapies

Given the multifactorial nature of heart failure, NRF2 modulators are unlikely to serve as monotherapies but rather as potent adjuncts. There is a strong theoretical rationale for combination therapies. Potential synergy with sodium-glucose cotransporter-2 (SGLT2) inhibitors is particularly notable, as SGLT2 inhibitors improve mitochondrial energetics and activate AMPK/SIRT1 pathways that converge with NRF2 signaling to enhance metabolic flexibility [68,69]. Furthermore, combining NRF2 activators with neurohormonal blockade-such as angiotensin receptor neprilysin inhibitors (ARNIs) or mineralocorticoid receptor antagonists (MRAs)-could hypothetically provide additive benefits by simultaneously targeting fibrosis, hemodynamic load, and oxidative stress [69]. However, considering the complexity of heart failure polypharmacy, extreme vigilance is required. Clinical studies must rigorously assess whether pharmacological redox modulation antagonizes the beneficial, physiological inflammatory signaling required for tissue repair [15].

#### 4.2.3. Biomarkers for Patient Stratification

The failure of broad-spectrum antioxidant trials suggests that a “one-size-fits-all” approach is likely ineffective. The development of validated biomarkers is proposed as a prerequisite for precision patient stratification, aiming to identify specific sub-populations with an “NRF2-deficient” phenotype who are most likely to benefit from therapy. Future diagnostic strategies should explore the utility of creating a composite “redox signature” by integrating multi-omics data. This would include transcriptomic profiling of NRF2 target genes (e.g., NQO1, GCLM) in peripheral blood mononuclear cells, proteomic analysis of oxidative damage markers (e.g., nitrotyrosine), and metabolomic assessment of the GSH/GSSG ratio [46]. Additionally, epigenetic screening for NRF2 promoter methylation could identify patients with acquired resistance to endogenous activation. While promising conceptually, the predictive value of these biomarkers requires prospective validation in large-scale clinical cohorts before they can guide treatment decisions [46,70,71], as summarized in Table 1.

## 5. Conclusions and Future Perspectives

### 5.1. NRF2 as a Central Regulator of Cardiovascular Redox Homeostasis

In summary, the NRF2-KEAP1 axis functions not merely as a responsive antioxidant switch but as a central integration hub governing the structural and metabolic fidelity of the cardiovascular system. As detailed comprehensively in this review, the protective scope of NRF2 extends far beyond simple radical scavenging to orchestrate a sophisticated, multi-layered defense strategy. At the organelle level, NRF2 is indispensable for mitochondrial integrity, preserving bioenergetics through the upregulation of biogenesis factors like PGC-1α and preventing lethal necrosis by inhibiting the opening of the mPTP. At the cellular level, emerging evidence highlighted in this paper underscores its pivotal role in suppressing ferroptosis, a lipid-peroxidation-dependent cell death, via the transcriptional activation of the SLC7A11-GPX4 axis. Furthermore, within the extracellular matrix, NRF2 serves as a critical brake on pathological remodeling by antagonizing the TGF-β/Smad signaling cascade, thereby limiting myofibroblast differentiation and interstitial fibrosis [15]. Consequently, extensive preclinical data confirm that NRF2 is a central, druggable node capable of determining the trajectory of heart failure, acting as a bulwark against the diverse insults of ischemia, pressure overload, and metabolic toxicity.

### 5.2. The Paradox of Acquired Insufficiency and Reductive Stress

Despite this robust protective potential, the translation of NRF2 biology into clinical practice is complicated by the distinct pathophysiological paradoxes discussed in this review. A primary challenge remains the phenomenon of “acquired NRF2 insufficiency” observed in the failing human heart. As we have examined, this loss of reserve capacity is driven by a convergence of epigenetic silencing via promoter methylation, aberrant degradation mediated by GSK-3β, and the disruption of the p62-dependent autophagy feedback loop. This downregulation renders the chronic heart failure myocardium critically vulnerable to oxidative damage. Conversely, the opposing end of the spectrum presents the risk of “reductive stress”. Unchecked, supranormal activation of NRF2 can annihilate physiological ROS signaling required for protein folding and angiogenesis, potentially leading to protein aggregation cardiomyopathies or maladaptive vascular remodeling as seen in pulmonary hypertension. Therefore, future research directions must pivot from simply asking how to activate NRF2, to elucidating how to restore its physiological range. It is essential to decipher the specific epigenetic and post-translational checkpoints that lock the pathway in a silenced state during chronic remodeling, as reversing these molecular brakes offers a more rational strategy than forcing activation in a dysregulated system.

### 5.3. The Evolution Toward Precision Therapeutic Modulation

The therapeutic landscape is consequently undergoing a necessary evolution from systemic, indiscriminate induction toward precision redox medicine. The history of NRF2 pharmacology, particularly the lessons learned from the Bardoxolone methyl trials, illustrates the dangers of broad-spectrum electrophilic activation, where off-target engagement of pathways like endothelin-1 can precipitate fluid retention and clinical decompensation. The future of NRF2-based therapeutics lies in the refinement of intervention strategies that decouple cardioprotection from these systemic liabilities. This review highlights the promise of next-generation PPI inhibitors, which offer non-covalent, highly specific displacement of NRF2 from KEAP1, minimizing the side effects of earlier agents. Moreover, the integration of advanced delivery systems, such as cardiac-homing nanoparticles and cardiotropic AAV9 gene therapy vectors, represents a critical frontier to achieve tissue-specific modulation. Ultimately, the successful clinical implementation of these modulators will depend on the concurrent development of validated multi-omics biomarkers to identify patient subgroups with a demonstrable “redox-deficient” phenotype. By harmonizing these pharmacological, technological, and diagnostic advancements, it is possible to harness the potent defense mechanisms of the NRF2-KEAP1 axis to arrest the progression of cardiovascular disease while navigating the complex boundary between essential physiological signaling and pathological stress.

## Figures and Tables

**Table 1:** Pharmacological Strategies of NRF2 in Cardiovascular Drug Development.

Category	Agent	Mechanism	Preclinical Evidence	Clinical Development Status	Key Findings	Limitations	Future Directions	References
Covalent Electrophilic Modulators	Sulforaphane (SFN)	Covalent modification of KEAP1 cysteine residues (C151, C273, C288) → NRF2 stabilization	I/R injury: ↓ infarct size (~30–40%) DCM models: ↓ fibrosis, ↑ diastolic function TAC models: ↓ hypertrophy	Phase I/II (non-cardiac) No dedicated CV trials	Well-tolerated in humans (Phase I) Suboptimal PK: short t½ (~2 h), low bioavailability	Poor oral bioavailability Rapid metabolism (GST conjugation) Difficulty achieving sustained cardiac exposure	Nanoparticle encapsulation Prodrug development Combination with PK enhancers	[54,55,56]
	Bardoxolone methyl (CDDO-Me)	Potent electrophilic triterpenoid; KEAP1 modification + direct mitochondrial effects	I/R injury: ↓ infarct size, ↓ mPTP opening Diabetic models: ↑ eGFR, ↓ albuminuria	Phase III (BEACON)-TERMINATED CKD + T2DM patients (n = 2185)	↑ eGFR (+5.5 mL/min/1.73 m^2^) vs. placebo Early termination: ↑ HF hospitalizations (HR 1.83, 95% CI 1.32–2.55)	HF events concentrated in high-risk patients (baseline BNP > 200 pg/mL, prior HF Hx) Mechanism: fluid retention (ET-1 ↑, Na^+^ retention), not direct cardiotoxicity Systemic, non-selective activation	Patient stratification: exclude high-risk HF patients Lower, pulsatile dosing regimens Cardiac-restricted delivery	[57,58,59,60]
	Dimethyl fumarate (DMF)	Electrophilic succination of KEAP1; also activates HCAR2	I/R injury: ↓ oxidative stress MS models	Approved for MS; off-label use explored	FDA-approved safety profile (MS)	GI side effects (flushing, diarrhea) Limited cardiac-specific data	Repurposing trials for acute MI (peri-PCI administration)	[60,61]
Protein–Protein Interaction (PPI) Inhibitors	Small-molecule PPI inhibitors (e.g., K67, CPUY192018)	Non-covalent, competitive disruption of KEAP1 Kelch domain–NRF2 Neh2 (ETGE/DLG motifs) binding	In vivo NRF2 activation (liver, kidney) Renal fibrosis models: ↓ TGF-β/Smad signaling Cardiac fibrosis (preliminary): ↓ myofibroblast differentiation	Preclinical	High specificity: no off-target cysteine reactivity Dose-dependent NRF2 activation with wider therapeutic window Demonstrated oral bioavailability (rodents)	Still subject to systemic risks of chronic NRF2 hyperactivation (oncogenic liability, metabolic reprogramming) Long-term safety data lacking	First-in-human trials (Phase I PK/PD studies) Rigorous dose-finding to avoid supraphysiological activation Development of cardiac-tropic analogs	[15,62,63]
	Peptide-based PPI inhibitors (e.g., cyclic ETGE-mimetic peptides)	High-affinity mimicry of NRF2 Neh2 domain; competitively displaces endogenous NRF2 from KEAP1	Proof-of-concept in cell culture	Early preclinical	Exquisite binding specificity (nM affinity) Rational design based on crystal structures	Poor cell permeability (requires CPP conjugation or lipid modification) Proteolytic instability (requires cyclization/D-amino acids) High production cost	Stapled peptides with improved PK Nanocarrier-mediated delivery Potential for inhaled/IV formulations (acute settings)	[15,62,63]
Targeted Delivery and Regulated Gene Expression	Cardiac-homing nanoparticles (e.g., ROS-responsive liposomes, polymeric NPs)	Encapsulation of NRF2 activators + functionalization with cardiac-targeting ligands (e.g., ischemia-targeting peptides, CREKA)	Proof-of-concept in cell culture	Proof-of-concept	Reduced systemic toxicity	Scalability and GMP manufacturing challenges Potential immunogenicity (PEGylation, complement activation) Regulatory pathway unclear (drug-device combination)	Translation to large animal models (pigs, dogs) Development of clinically compatible formulations IND-enabling toxicology studies	[64,65]
	AAV9-mediated NRF2 gene delivery	Cardiotropic AAV serotype → cardiac-specific NRF2 overexpression (±inducible promoters: α-MHC, Tet-on)	I/R injury: ↓ infarct size, ↑ capillary density TAC models: ↓ pathological remodeling DCM models: ↑ mitochondrial biogenesis	Preclinical	Durable expression	Neutralizing antibodies (~40–60% human prevalence against AAV9) → limits repeat dosing Risk of constitutive hyperactivation if promoter leaky Vector immunogenicity (innate/adaptive responses)	Capsid engineering (immune-evasion variants) Pre-screening for anti-AAV Ab; immunosuppression protocols “Hit-and-run” gene editing (CRISPR activation of endogenous NFE2L2)	[66]
	Exosome-delivered NRF2 mRNA/protein	Endogenous nanovesicles loaded with NRF2 cargo → fusion with target cells	I/R injury: ↓ oxidative damage, ↑ post-MI function	Early preclinical	Biocompatible, low immunogenicity	Production scalability Cargo loading efficiency Short-lived effect (requires repeat dosing)	Engineering of “super-exosomes” with enhanced homing/loading Autologous sources (iPSC-derived)	[66]
Combination Therapies	NRF2 activator + SGLT2 inhibitor (e.g., SFN + Empagliflozin)	Convergent mechanisms: ↑ mitochondrial health, ↓ NLRP3 inflammasome, ↑ FAO	Diabetic models: synergistic ↓ in fibrosis, ↑ EF SGLT2i alone activates NRF2 (via AMPK, SIRT1)	Preclinical	SGLT2i is standard of care in HFrEF/HFpEF → low barrier to combination trials	Potential for over-suppression of physiological ROS signaling Drug-drug interaction studies needed	Phase II clinical trial design: NRF2 activator as add-on to SGLT2i in HFpEF patients with high oxidative stress biomarkers	[68,69]
	NRF2 activator + ARNI or MRA	Multi-pathway targeting: redox (NRF2) + neurohormonal (RAAS) + fibrotic pathways		Conceptual			Mechanistic studies to define optimal sequencing and dosing	[69]
Biomarker-Guided Precision Medicine	Composite “Redox Signature” for patient stratification	Multi-omics integration: Transcriptomics (NQO1, GCLM expression in PBMCs) Proteomics (plasma HO-1, SOD2) Metabolomics (GSH/GSSG ratio) Epigenomics (NFE2L2 promoter methylation)	Validation in HF cohorts: “NRF2-deficient” phenotype correlates with worse outcomes	Biomarker discovery	Identifies patients most likely to benefit Enables precision dosing	Assay standardization and reproducibility Cost and accessibility of multi-omics platforms Need for prospective validation in RCTs	Companion diagnostic development Integration into clinical trial design Point-of-care redox biomarker devices	[22]

Abbreviations: AAV9, adeno-associated virus serotype 9; ARNI, angiotensin receptor-neprilysin inhibitor; BNP, B-type natriuretic peptide; CDDO-Me, bardoxolone methyl (2-cyano-3,12-dioxooleana-1,9(11)-dien-28-oic acid methyl ester); CPP, cell-penetrating peptide; CREKA, Cys-Arg-Glu-Lys-Ala (ischemia-targeting peptide); DCM, diabetic cardiomyopathy; DMF, dimethyl fumarate; EF, ejection fraction; eGFR, estimated glomerular filtration on rate; ET-1, endothelin-1; FAO, fatty acid oxidation; GI, gastrointestinal; GMP, good manufacturing practice; GSH/GSSG, reduced/oxidized glutathione ratio; HFpEF, heart failure with preserved ejection fraction; HFrEF, heart failure with reduced ejection fraction; HO-1, heme oxygenase-1; I/R, ischemia/reperfusion; IND, investigational new drug; iPSC, induced pluripotent stem cell; MI, myocardial infarction; MRA, mineralocorticoid receptor antagonist; mPTP, mitochondrial permeability transition pore; MS, multiple sclerosis; Neh2, NRF2-ECH homology 2 domain; NP, nanoparticle; NQO1, NADPH quinone dehydrogenase 1; PCI, percutaneous coronary intervention; PK, pharmacokinetics; PD, pharmacodynamics; PPI, protein–protein interaction; RCT, randomized controlled trial; SFN, sulforaphane; SGLT2i, sodium-glucose cotransporter-2 inhibitor; SOD2, superoxide dismutase 2; T2DM, type 2 diabetes mellitus; TAC, transverse aortic constriction; Tet-on, tetracycline-inducible system; TGF-β, transforming growth factor-beta. Key to Symbols: ↑ = Increase/upregulation; ↓ = Decrease/downregulation; ~ = Approximately; vs. = Versus; t½ = Half-life. Clinical Trial Status Legend: Preclinical: In vitro/in vivo animal studies; Phase I: First-in-human safety/PK studies; Phase II: Proof-of-concept efficacy trials; Phase III: Large-scale RCTs for registration; Approved: Regulatory approval obtained (for specific indication); Terminated: Trial stopped due to safety/futility.

## Data Availability

No new data were created or analyzed in this study. Data sharing is not applicable to this article.
